# The impact of frequency-specific music stimulation on consciousness in patients with disorders of consciousness

**DOI:** 10.3389/fneur.2025.1506261

**Published:** 2025-02-25

**Authors:** Haitao Yang, Wenhao Huang, Wei Wen, Shoufeng Long, Yujie Zhang, Xiangfeng Chi, Daihong Luo

**Affiliations:** ^1^The First Affiliated Hospital of Guangzhou University of Chinese Medicine, Guangzhou, China; ^2^The Third Affiliated Hospital of Sun Yat-sen University, Guangzhou, China; ^3^Guangzhou University of Chinese Medicine, Guangzhou, China; ^4^The Second Clinical College of Guangzhou University of Chinese Medicine, Guangzhou, China; ^5^Guangdong No. 2 Hospital of Traditional Chinese Medicine, Guangzhou, China; ^6^Beijing University of Chinese Medicine Shenzhen Hospital (Longgang), Shenzhen, China

**Keywords:** music stimulation, consciousness, disorders of consciousness, frequency-specific, fNIR

## Abstract

**Objective:**

This study aimed to evaluate the effects of frequency-specific music stimulation on the awareness and brain connectivity in patients with disorders of consciousness (DOC).

**Methods:**

A total of 25 DOC patients were exposed to auditory stimulation through music at varying frequencies (low: <0.5 Hz, middle: 0.5 Hz–3.5 kHz, high: >3.5 kHz). Brain responses were assessed using Functional Near-Infrared Spectroscopy (fNIRS) to monitor objective markers of brain activity. The analysis focused on effective connectivity and coupling strength (CS) values in response to different frequency stimulations, targeting regions such as the motor and somatosensory cortices.

**Results:**

The mean age of the patients was 49.4 years, with an average coma duration of 1.96 months. While no significant differences were observed in general brain arousal across different frequency stimuli, notable differences in effective connectivity were identified. High-frequency stimulation resulted in significantly higher CS values in the right primary motor cortex (*p* < 0.05), while middle-frequency stimulation showed significant effects in the right primary somatosensory cortex (*p* = 0.016).

**Conclusion:**

The findings suggest that middle- and high-frequency music stimulation may enhance effective connectivity in specific brain regions, potentially contributing to the rehabilitation of DOC patients. These results indicate that frequency-specific music could stimulate motor networks and areas associated with autobiographical memory, highlighting its therapeutic potential in promoting awareness in this patient population.

## Introduction

Disorders of consciousness (DOC) are states of unconsciousness caused by severe brain trauma, coma, unresponsive wakefulness syndrome (UWS) and Minimally conscious state (MCS). These conditions are characterized by decreased arousal and alterations in the content of consciousness (1). An article provides the first estimates of the incidence and prevalence of coma in the United Kingdom and the United States through a crowdsourcing approach. According to the study, the annual incidence rate was found to be 135/100,000 in the UK and 258/100,000 in the US (2).

The treatment of consciousness disorders presents significant challenges in clinical practice. Current treatment methods consist of pharmacotherapy, hyperbaric oxygen therapy, neuromodulation therapy, traditional Chinese medicine therapy, and comprehensive rehabilitation therapy (3, 4). However, sufficient and conclusive evidence regarding their efficacy is still lacking. In recent years, music therapy has arisen as a possible adjunctive therapy for a variety of diseases (5). Previous research involving 29 studies and 775 patients with brain injuries found that listening to music enhanced gait, upper limb function duration, communication outcomes, and quality of life after stroke (6). Another study found that music provides a distinct sensory stimulus, with EEG performance demonstrating more pronounced responses than non-musical controls (7–9). Research on musicalized name arousal in patients with consciousness disorders revealed sustained responses of the cerebral hemispheres to musical stimulation. In addition, familiar sounds from family members increased brain responses to acoustic stimuli in patients with consciousness disorders, whereas combining musical melodies led to more active responses in brain regions associated with arousal. It was also discovered that music therapy can aid in the recovery of consciousness in patients with consciousness disorders (10).

Traditional and effective rehabilitation techniques such as music therapy have demonstrated positive effects on consciousness recovery. Due to the vast variety of available music and individual preferences, however, there is still ample room for exploration in selecting the most stimulating music for an individualized music therapy program for different patients, as well as understanding its effects on the brain network of patients with consciousness disorders (10–13). The frequency-specific nature of these effects is crucial to our study, as it supports the hypothesis that different sound frequencies might differentially influence cognitive and motor responses, which could be pivotal for applications in therapeutic or enhancement contexts.

The idea that specific sound frequencies directly impact cognitive and motor responses is well-established. However, the specific mechanisms through which sound influences cognitive processes remain unclear. Two important studies from the cognitive science literature provide key insights into this area. First, Pápai and Soto-Faraco demonstrated that sounds can boost visual awareness through attention, even without cross-modal integration. This effect highlights the significant role sound plays in enhancing the awareness of visual events (14). Second, Dresp-Langley and Monfouga showed that the amplitude of the effect of sound on visual detection, measured by motor response times, depends on the frequency of the sound (15). These findings underscore the frequency-specific nature of sound’s impact on cognitive processes, which is highly relevant to the present study’s exploration of sound’s role in motor responses. Recently, Xiao-Ying Zhang et al. (16) demonstrated that music with frequencies ranging from 20 to 20,000 Hz stimulated the primary auditory cortex with a broader spectrum of stimuli and more neural networks than music with frequencies ranging from 0 to 5,000 Hz. Moreover, Min Wu et al. demonstrated that rhythmic music-electrical TNS stimulation promoted rhythmic brain activity in patients with DOCs, particularly at certain stimulation frequencies, and that this enhanced awareness was functionally related to the increase in rhythmic brain activity (17). We contemplated, in light of this evidence, whether various music frequency stimulation influences the activation of neural networks and wakefulness in conscious patients. Therefore, we decided to investigate the effects of various music frequencies on patients in a conscious state in order to determine which music frequencies have the greatest therapeutic potential.

Functional near infrared spectroscopy (fNIRS) is a promising functional brain imaging technique that has been intensively researched for evaluating consciousness disorders caused by brain injuries (18–20). It has been utilized to detect residual consciousness in patients with consciousness disorders, to identify potentially conscious patients by detecting brain activity, and as a detection instrument in wake-promoting therapy (20). In one study, resting-state fNIRS was used to detect residual functional networks in DOC patients. The study discovered varying degrees of topological architecture loss in MCS and UWS, as well as significant differences in the regional nodal properties of BA10 between the two conditions (20, 21). Another study used an active command-driven motor imagery paradigm supported by fNIRS to detect residual consciousness in patients with protracted DOC. Active command-driven motor imagery confirmed the viability of using portable fNIRS technology to detect residual cognitive ability in patients with prolonged DOC. Imaging comatose patients is a complex and multifaceted process. In addition to fNIRS, several other imaging techniques are used to assess the brain status of comatose patients. For example, MRI provides high-resolution images of brain structures, which can help identify brain damage, tumors, or other abnormalities (22). EEG is a method of assessing neurological function by recording electrical activity in the brain, which is particularly useful for detecting seizures or brain dysfunction (23). And both of MRI and EEG are to to assess for covert awareness in an active paradigm. In our study, we will use fNIRS to investigate the state of consciousness and blood oxygen response in patients with DOC, with the goal of elucidating the differential effectiveness of various acoustic stimulation frequencies on consciousness disorders.

## Methods

### Participants

This experimental investigation was conducted in the Department of Rehabilitation Medicine at the Third Affiliated Hospital of Sun Yat-sen University, and it included 25 patients diagnosed with minimal consciousness disturbance following stroke. In this study, minimal consciousness disturbance refers exclusively to patients in a MCS, as defined by the Coma Recovery Scale-Revised (CRS-R) and Glasgow Coma Scale (GCS). Patients in a coma or in an UWS were excluded from the study to ensure a homogenous sample of MCS patients who retain some level of awareness and potential for therapeutic engagement. This focus allows for a targeted evaluation of the effects of frequency-specific music stimulation on patients with residual consciousness.

The purpose of the study was to examine the effects of a specific intervention on stroke patients with minimal consciousness impairment. The inclusion and exclusion criteria were established to guarantee the study participants’ eligibility and suitability. The experimental methods were approved by the Ethics Committee of the Third Hospital of Sun Yat-sen University Hospital and conformed to the 1975 Declaration of Helsinki (revised in 2008). The registration number was ChiCTR2300071904, which was verified by the Chinese Clinical Trial Registry.

The following were the inclusion criteria for patients with minimal post-stroke impairment of consciousness: (a) patients met the diagnostic criteria for cerebrovascular disease established by the Fourth National Cerebrovascular Conference, and the diagnosis was confirmed by imaging such as craniocerebral CT or MRI (24); (b) patients were in a MCS, as determined by the CRS-R and GCS scores between 3 and 8; (c) patients exhibited residual consciousness, defined as inconsistent but reproducible responses to external stimuli; (d) patients were not using centrally-activated medication or sedation; (e) patients were not hearing impaired prior to the illness, allowing for participation in auditory stimulation tasks.

The following criteria determined exclusion: (a) patients did not have severe speech and cognitive dysfunction prior to the illness; (b) patients with a history of cognitive dysfunction; (c) patients with serious complications such as cardiorespiratory, hepatic, or renal insufficiency; (d) patients with irreversible visual and auditory dysfunction; and (e) patients who withdrew from the study for any reason.

To ensure consistency and limit confounding variables, patients in a coma or UWS were excluded. Additionally, the time since stroke onset was limited to no more than 6 months. This limit was based on evidence suggesting that earlier intervention is more likely to influence neural plasticity and improve recovery outcomes. The inclusion criteria were designed to select a homogenous population of MCS patients with potential for therapeutic responsiveness.

### Procedures

#### Musical stimulation modalities

Each patient received musical stimuli at three different frequencies: low frequency (below 0.5 Hz), mid-frequency (0.5 Hz to 3.5 kHz) and high frequency (above 3.5 kHz). The order of these stimuli was randomized. Each stimulus lasted 9 min, with a 9-min interval between each set of music, for a total experimental duration of 54 min. Mozart’s Concerto No. 2 in E major was chosen, with a duration of 224 s and a tempo of approximately 98 beats per minute. The music was played at a constant tempo with a constant rhythm. Musical frequencies were adjusted using specialized music software to achieve the desired frequency modifications.

Using specialized music software, the music frequencies were altered to accomplish the intended frequency modifications. Frequencies of 3.5 kHz or higher were amplified by a factor of two for the high-frequency group. In the low-frequency group, low and mid-frequency frequencies below 0.5 Hz were amplified by a factor of two. Lastly, the frequency band between 0.5 Hz and 3.5 kHz was amplified by a factor of two in the mid-frequency group. Prior to music therapy, the patient’s condition was evaluated using the Coma Scale, and during music therapy, the patient’s condition was continuously monitored in real time using fNIRS.

### Outcome measurement

#### Scale assessment

Before administering musical stimulation, the subjects’ level of consciousness will be evaluated using the Glasgow Coma Scale (GCS) and the Coma Recovery Scale, Revised (CRS-R). The GCS is a widely utilized clinical instrument for assessing consciousness impairment in comatose patients. It includes the eye-opening response, the verbal response, and the motor response. Maximum score of 15 indicates normal consciousness, while scores between 12 and 14 indicate modest impairment, 9 to 11 indicate moderate impairment, and 8 or less indicate a moribund state. A lower score indicates a more severe level of consciousness impairment.

While it remains a cornerstone of clinical practice, recent studies, including a review by Zafar et al. (36), have highlighted several limitations of GCS as a consciousness assessment tool (25). These limitations include its heavy reliance on motor and verbal responses, which may underestimate residual consciousness in patients with severe motor or communication impairments, such as those in a MCS. To address these concerns in the current study, GCS was complemented with CRS-R, which provides a more comprehensive assessment of consciousness by incorporating auditory, visual, motor, and communication subscales.

The Coma Recovery Scale-Revised (CRS-R), also known as the Coma Recovery Scale-Revised, was introduced in 2004 and has since become the behavioral assessment scale of choice for diagnosing patients with disorders of consciousness (DOC) (26, 27). Six subscales comprise the CRS-R: auditory, visual, motor, verbal, communication, and arousal. The highest possible score on the CRS-R is 23, with higher scores indicating less severe consciousness disturbances. The CRS-R scoring criteria for patients in a vegetative state (VS) are: auditory score 2, visual score 1, motor score 5, verbal response score 2, communication score = 0, and arousal score 2. The CRS-R evaluation criteria for patients in a minimally conscious state (MCS) are as follows: auditory score > 2, visual score > 1, motor score > 2, verbal response score > 2, communication score > 0, or arousal score > 2.

The GCS and CRS-R assessments were performed by a single trained evaluator to ensure consistency across all patients.

#### Functional near–infrared spectroscopy measurement

**Figure 1:**
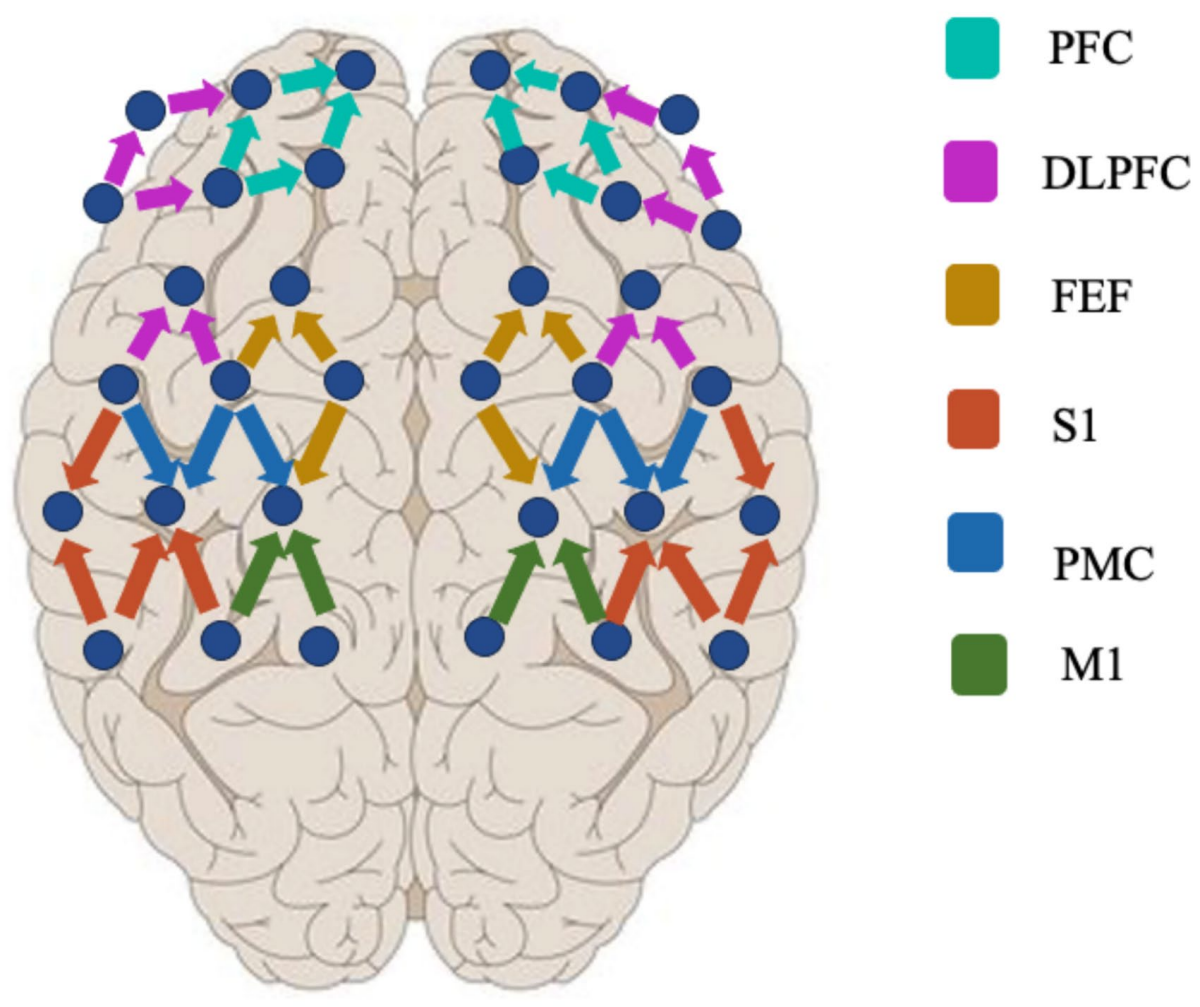
Distribution of fNIRS probes.

The fNIRS data acquisition experiments were performed using a multichannel NIR system (Nirsmart, Danyang Huichuang Medical Equipment Co., Ltd., China) to record changes in oxygenated (HbO) and deoxygenated (HbR) signals (35). The experimental design used 15 sources and 16 detectors for a total of 48 active channels (the device contains 24 sources and 24 detectors for more than 80 active channels), with a channel spacing of 3 cm (Figure 1). The reference for the international 10–20 localization system included frontal, parietal, and temporal lobe regions. The light source probes were at 730 nm and 850 nm, and the detectors were very sensitive avalanche diode APDs. To organize the emitter and collector, a head cap was used. Before the experiment, a pre-test was performed to check that all channels were used appropriately.

#### Wavelet amplitude

Wavelet amplitude (WA) analysis is a signal processing technique used to analyze the time-frequency characteristics of fNIRS data. The analysis involves decomposing the fNIRS signal into different frequency bands and examining the amplitude changes within each band over time. We used a specific type of mother wavelet for the continuous wavelet transform (WT), chosen to have a specific center frequency and number of oscillations. The wavelet amplitude is calculated by taking the absolute value or square of the wavelet coefficients for each frequency component at each time point. This analysis helps to identify changes in brain activity over time within specific frequency bands, providing important insights into the brain’s response to external stimuli.

It involves decomposing the fNIRS signal into different frequency bands and examining the amplitude variations within each band over time. To perform WA analysis on fNIRS data, the following steps are typically followed. Specific filters were used in the preprocessing of the fNIRS data to remove noise and artifacts. The removal of artifacts is accomplished through a combination of visual inspection and automated methods, ensuring the accuracy and reliability of the data. The preprocessed fNIRS signal is then subjected to the continuous wavelet transform (WT). The WT is a transformation method of time series from time domain to frequency domain that decomposes the signal into different frequency components at different time points. It provides a time-frequency representation of the signal. Once the wavelet transform is applied, the wavelet coefficients are obtained for each frequency component at each time point. The WA represents the magnitude of the signal in each frequency band at different time points. The amplitude is calculated by taking the absolute value or squaring the wavelet coefficients. The WA obtained in the previous step are used to examine the time-frequency characteristics of the fNIRS signal. This analysis helps identify changes in brain activity over time within specific frequency bands.

The WA analysis of fNIRS data provides valuable insights into the temporal dynamics of brain activity in comatose patients. It can help identify changes in different frequency bands, such as oscillatory activity related to specific brain regions or cognitive processes. By monitoring these changes, clinicians and researchers can better understand the brain’s response to external stimuli and potentially predict or assess a patient’s wakefulness-promoting effects for specific music.

#### Effective connectivity

Effective connectivity (EC) refers to the influence that one brain region exerts over another, allowing for the exchange of information and communication within the brain network. It provides insights into the directional relationships and interactions between different brain regions. Granger Causality Analysis (GCA) is a statistical approach used to infer the directionality of causal interactions between brain regions. It assesses whether the past activity of one region improves the prediction of the future activity in another region, providing insights into the effective connectivity within the brain network. To apply GCA to fNIRS data and investigate effective connectivity, the following steps are typically followed. The raw fNIRS data is preprocessed to remove noise and artifacts. This may involve filtering the signal, motion correction, and baseline correction. Based on prior knowledge or research hypotheses, specific brain regions of interest (ROIs) are defined. These ROIs are typically chosen based on anatomical or functional considerations. The fNIRS data from the selected ROIs is extracted, resulting in time series data for each ROI. These time series represent the measured hemodynamic responses in each ROI over time. A multivariate autoregressive model, such as a Vector Autoregressive (VAR) model, is fitted to the time series data from the ROIs. The VAR model estimates the causal interactions and directionality between the ROIs by modeling the dependencies among the time series. The GCA are computed based on the fitted VAR model. Granger causality provides a quantitative measure of the causal influence one ROI has on another ROI. It assesses whether the inclusion of past activity from one ROI improves the prediction of the future activity in another ROI.

Dynamic Bayesian inference (DBI) is based on prior knowledge used to estimate current or posterior model parameters or uses the acquired knowledge to improve the reasoning which could extract the optimal set of parameters for describing the model. The properties of a network of N-coupled periodic oscillators can be analyzed based on its phase dynamics. A system of N stochastic differential equations varying with time was established according to the original signal phase information obtained by WT. In this study, we established coupling functions based on coupled-phase-oscillator model in a pairwise manner and utilized DBI theory to infer directed coupling interactions for all possible pairs of channels. The directed coupling strength $\Phi_{i\to j}$ represents the influence of oscillation i on oscillatorj and $\Phi_{j\to i}$ represents the influence of oscillation j on oscillatori. Coupling strength of all possible pairs of channels were determined by DBI. A surrogate test was then applied to ascertain whether the detected coupling parameters are genuine or spurious. The directed coupling interactions were derived from all possible pairs of the 100 surrogate signals for every two channels based on coupled-phase-oscillator model and DBI theory. The directed coupling strength of the real signals was considered significant if the value was 2 standard deviations above the surrogate means.

By applying GCA to fNIRS data, researchers can identify the directionality of causal interactions between specific brain regions. This allows them to infer the effective connectivity within the brain network and understand how information flows and is exchanged between different regions. By applying EC to fNIRS data, researchers can identify the directionality of causal interactions between specific brain regions. This allows them to infer the EC within the brain network and understand how information flows and is exchanged between different regions.

### Statistical analysis

The Kolmogorov–Smirnov test and the Levene test were performed in this study to ensure that the obtained values met the assumption required by the analysis of variance (ANOVA) analysis. One-way repeated ANOVA was performed to assess the main differences in the WA and EC values. The statistical significance was set to *p* < 0.05 with Bonferroni-correction for pair-wise comparisons.

## Results

Twenty-five patients were included in the experiment’s data analysis. Age, gender, and time of stroke onset were collected for each patient. The preponderance of the patients had either brain hemorrhage or cerebral infarction, with a mean age of 49.4 years. Using the GCS with a mean score of 4.2 (SD = 1.8) and the CRSR with a mean score of 2.4 (SD = 2.8), the degree of consciousness impairment was determined.

To ensure accurate classification of patients as having DoC, all participants underwent comprehensive neuroimaging assessments, including CT or MRI. These scans were reviewed to confirm that the location of the stroke or brain injury involved regions known to be physiologically associated with DoC, such as the thalamus, brainstem, or extensive cortical and subcortical areas. Detailed information on the lesion location for each patient is provided in the Table 1.

**Table 1:** Characteristics of patients.

Patients	Causes	Stroke location	DoC relevance	Age	Onset time	GCS score	CRSR score
Patient 1	Ischemic	Vagus	Yes	31	3 m	3	2
Patient 2	Infarction	Cerebral cortex	Yes	56	1 m	3	0
Patient 3	Ischemic	Vagus	Yes	54	1 m	3	5
Patient 4	Infarction	Vagus	Yes	24	1 m	3	0
Patient 5	Ischemic	Cerebral cortex	Yes	28	1 m	6	8
Patient 6	Autoimmune encephalitis	Cerebral cortex	Yes	65	1 m	4	5
Patient 7	Infarction	Cerebral cortex	Yes	68	2 m	3	0
Patient 8	Infarction	Cerebral cortex	Yes	58	1 m	6	7
Patient 9	Infarction	Brainstem	Yes	57	4 m	3	0
Patient 10	Ischemic	Cerebral cortex	Yes	73	3 m	8	8
Patient 11	Infarction	Cerebral cortex	Yes	45	5 m	3	0
Patient 12	Hypoxic–ischemic	Vagus	Yes	38	1 m	4	1
Patient 13	Ischemic	Cerebral cortex	Yes	25	0.3 m	4	2
Patient 14	Hemorrhage	Cerebral cortex	Yes	29	0.5 m	4	2
Patient 15	Hemorrhage	Vagus	Yes	38	0.3 m	3	0
Patient 16	Hemorrhage	Brainstem	Yes	42	0.5 m	6	3
Patient 17	Brain occupying lesions	Brainstem	Yes	53	1 m	5	2
Patient 18	Infarction	Brainstem	Yes	51	1 m	3	0
Patient 19	Infarction	Vagus	Yes	58	1 m	3	0
Patient 20	Viral encephalitis	Cerebral cortex	Yes	65	4 m	3	0
Patient 21	Infarction	Cerebral cortex	Yes	63	0.25 m	6	3
Patient 22	Hemorrhage	Cerebral cortex	Yes	68	2 m	3	4
Patient 23	Infarction	Vagus	Yes	41	0.7 m	3	0
Patient 24	Infarction	Cerebral cortex	Yes	46	0.6 m	8	8
Patient 25	Hemorrhage	Vagus	Yes	59	1 m	3	0

### Cortical activation patterns

Using a multichannel fNIRS system, the cerebral hemodynamics of the participants were measured during specific conditions (resting state and music sessions of varying frequencies). Dorsolateral prefrontal cortex (DLPFC), prefrontal cortex (PFC), premotor cortex (PMC), frontal eye field (FEF), primary somatosensory cortex (S1), and primary motor cortex (M1) were regions of interest (ROIs). For all patients with left-sided lesions, the fNIRS data were inverted along the midsagittal plane so that the lesioned side corresponded to the right hemisphere.

**Figure 2:**
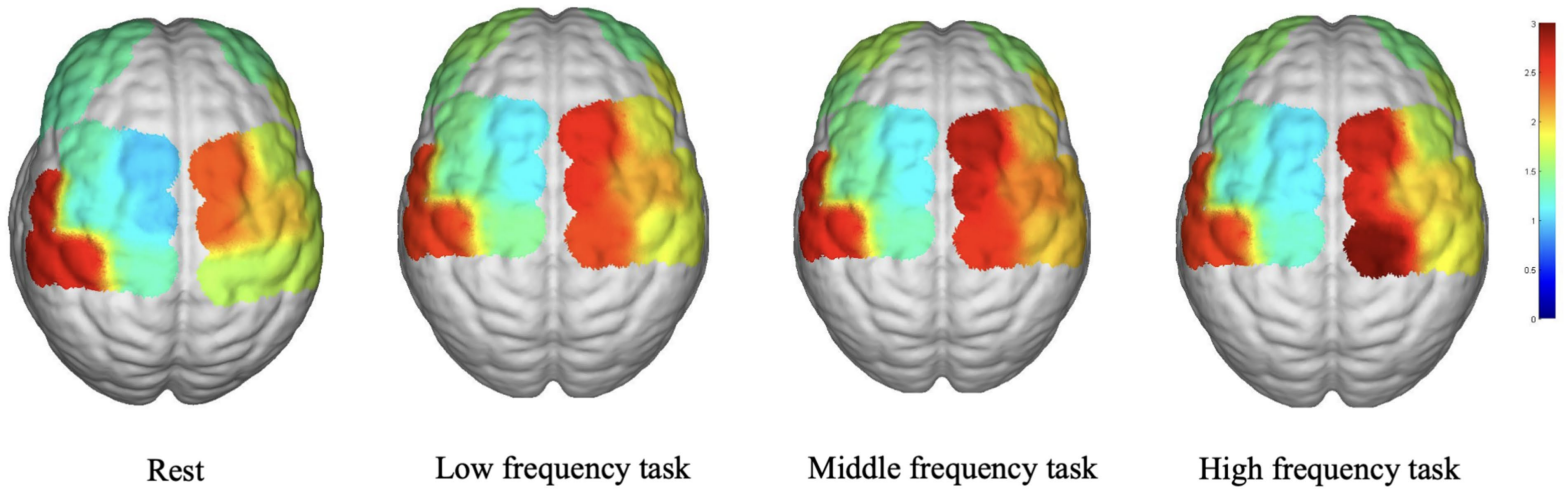
The result of WA.

Figure 2 depicts the average WA values in various brain regions during the various task states. In the LPFC, LDPLFC, LFEF, RFEF, and RS1 regions, the three task states exhibited an upward trend in WA. In each cohort, the LM1, RPFC, LPMC, RDLPFC, and RPMC regions exhibited a similar pattern. In addition, the average WA values in the LS1 region exhibited an upward trend during the resting state. Despite the fact that some brain regions displayed either increasing or decreasing trends, there were no statistically significant differences between the three task states and the rest state.

### Correlation analysis results

**Figure 3:**
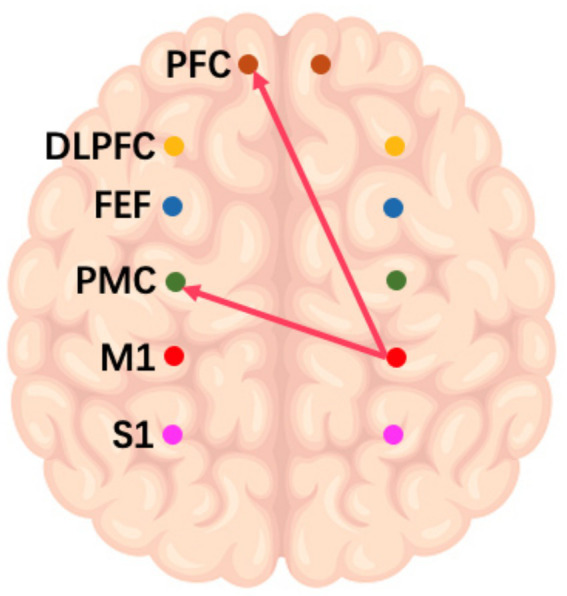
Diagram of a high-frequency music task. The arrows in the figure refer to the dominance of M1 in the right brain over PMC and PFC in the left brain during this task.

**Figure 4:**
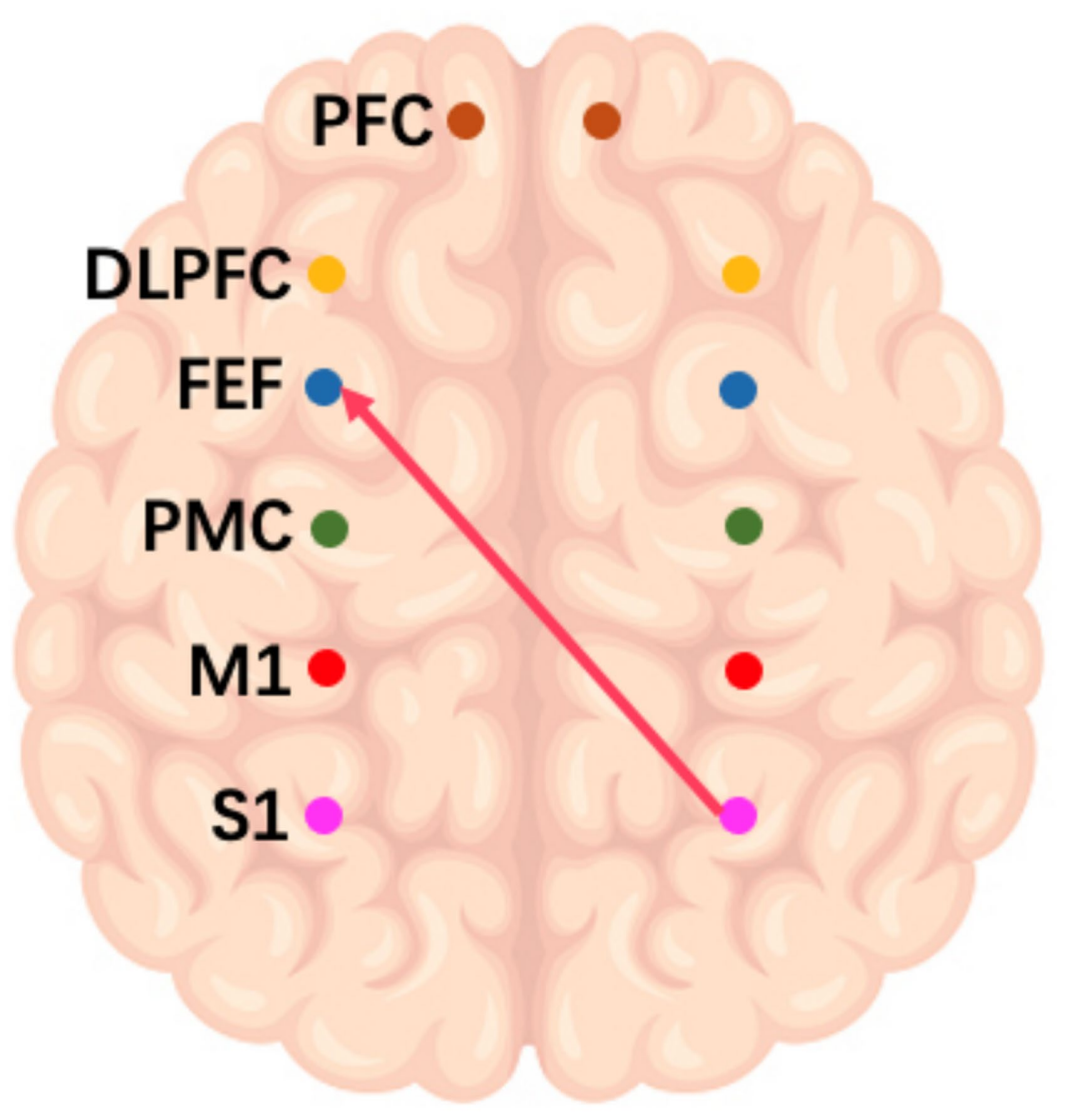
Schematic representation of the IF music task. The arrows in the figure refer to the dominance of S1 in the right brain over PMC and PFC in the left brain during this task.

On the frequency-specific coupling parameters for HbO2 signals, a repeated-measures ANOVA was conducted with the between-subject factor “group” and the within-subject factor “conditions.” During the high-frequency task, the analysis revealed that the coupling strength values of right M1 region exhibited a significant main effect of group in the effective connectivity from RM1 to LDLPFC (*p* = 0.046, *F* = 4.24) and from RM1 to LPFC (*p* = 0.016, *F* = 6.27) (Figure 3). In the middle-frequency task, the effective connectivity from RS1 to LFEF showed a significant main effect of group (p = 0.016, *F* = 7.56) (Figure 4). In the low-frequency state, neither condition nor interaction had a significant impact.

## Discussion

The results of this study show that specific sound frequencies have a significant effect on cognitive and motor responses. Our findings support the hypothesis that auditory stimuli can influence visual awareness and motor decision-making processes. These results are consistent with previous studies, such as those by Pápai and Soto-Faraco and Dresp-Langley and Monfouga, which highlight the role of sound in modulating awareness and motor responses.

Concerning the WA analysis, the increasing trend observed in the LPFC, LDPLFC, LFEF, RFEF, and RS1 during the three task states suggests that these brain regions may be more responsive and engaged during music stimulation across all frequencies. Multiple brain regions, including the LPFC, LDPLFC, LFEF, RFEF, and RS1, are implicated in cognitive and executive processes, attention, and emotional regulation, according to research (28–30). The similar trend observed in the LM1, RPFC, LPMC, RDLPFC, and RPMC suggests a common activation pattern across various music frequencies, suggesting their potential role in music processing and motor-related functions. The lack of significant differences in other brain regions between the three task states and the rest state suggests that these regions are less affected by the specific frequencies of music used in this study.

In addition to the WA analysis, the examination of frequency-specific coupling parameters offered additional insight into the effects of various musical conditions on brain connectivity. The significant main effects of group observed in the high and middle frequency conditions suggest that patients with impaired consciousness exhibit distinct responses. Patients had altered connectivity patterns between the right sensorimotor region and the left lateral prefrontal cortex. This finding suggests that high-frequency music may induce a dominant role for the right primary motor cortex (M1) in regulating the left prefrontal cortex (PFC), consistent with the known lateralization of brain functions (31). Collectively, these results underscore the frequency-dependent effects of music on brain networks and emphasize the need to consider specific frequency ranges when devising music-based interventions for patients with impaired consciousness.

Ischemic and hypoxic brain tissue necrosis is associated with the onset of impaired consciousness following a craniocerebral injury. This is due to the fact that cerebral ischemia and hypoxia can directly cause certain areas of the brain to cease functioning – for instance, the cerebral cortex cannot be activated effectively – and the basis of brain networks associated with consciousness is in an unbalanced state. The mechanisms that regulate consciousness stimulate the cerebral cortex, sustain arousal, and keep the body in a state of wakefulness. Activation of the cerebral cortex and regulation of brain–brain functional networks are therefore essential for arousal in disorders of consciousness. Studies (31) have demonstrated that resting-state functional connectivity within the default mode network (DMN) is attenuated and proportional to the degree of consciousness impairment in patients with atresia syndrome to coma. In addition, decreased functional connectivity within the DMN, particularly between the medial prefrontal cortex and the posterior cingulate cortex, can be used to predict the prognosis of patients with DOC (32). Therefore, interventions that enhance the neural network of the brain in patients with DOC are of utmost importance.

Area M1 is the superior nerve control center that regulates body movement. It plays a crucial role in the control of the body’s premeditated movements and voluntary movements. Area M1 receives and processes sensory information from the periphery before transmitting it to the basal ganglia. The cortico-basal ganglia-thalamo-cortical neural circuit processes, integrates, and transmits neural information, thereby influencing the body’s sporadic voluntary movements. Chronic disorders of consciousness (DOC) are characterized by a disconnection of the cortico-thalamo-cortical network, which correlates with behavioral responsiveness and level of consciousness. A study was conducted to determine the function of the M1 and PMC regions in patients with minimal consciousness disorder and unresponsive wakefulness syndrome (33, 34). The study revealed that motor unresponsiveness in some patients may be caused by a pure motor output failure, as in the functional locked-in syndrome, as opposed to a pre-motor-motor connectivity deficit. A study found that patients with Prolonged Disorders of Consciousness had damaged white matter fibers involved in voluntary motor control (9). The study utilized diffusion tensor imaging and diffusion tensor tractography to evaluate white matter injury in four patients with decreased consciousness due to a cerebral hemorrhage. In all four instances, the analysis identified significant frontal WMI as thinning and anatomical disruption. The frontal white matter tracts are a crucial component of the limbic system and ascending reticular activating system, and the frontal white matter index is associated with a low conscious level and cognitive dysfunction. Damage to the fiber tracts manifested as thinning, reduction in volume, or absence on tractography, accompanied by a decrease in the mean fractional anisotropy values of the affected side’s frontal white matter. Thus, our experiments revealed that listening to high-frequency music stimulates activity in the M1 region. While our findings suggest that listening to specific frequencies of music may influence brain activity and potentially support the re-establishment of neural network connections, it is important to note that no post-stimulation CRS-R assessments were conducted to directly assess consciousness changes. Bedside neurobehavioral assessments, such as the CRS-R, remain the gold standard for evaluating consciousness in patients with disorders of consciousness. Therefore, while our imaging results provide valuable insights into the potential mechanisms of action, future studies should include post-stimulation CRS-R evaluations to validate the clinical significance of these findings and assess whether music stimulation leads to measurable improvements in consciousness.

However, several limitations must be considered when interpreting these findings. First, while our study demonstrated that sound frequencies influence cognitive and motor responses, the mechanisms underlying these effects remain unclear. Future studies could benefit from incorporating neuroimaging techniques to better understand the neural pathways involved in these processes. Second, the sample size of our study was relatively small, which may limit the generalizability of our findings. Larger sample sizes and diverse populations could provide more robust evidence of the effects of sound on cognitive and motor responses. Third, the study was conducted in a controlled laboratory setting, which may not fully capture the complexity of real-world environments. Further research could explore how these effects translate to more ecologically valid contexts, such as in naturalistic settings or clinical populations.

Importantly, while our findings suggest that listening to specific frequencies of music may influence brain activity and potentially support the re-establishment of neural network connections, it is critical to note that no post-stimulation CRS-R assessments were conducted to directly evaluate changes in consciousness. Bedside neurobehavioral assessments, such as the CRS-R, remain the gold standard for evaluating consciousness in patients with DOC. Therefore, although our imaging results provide valuable insights into the potential mechanisms of action, future studies should include post-stimulation CRS-R evaluations to validate the clinical significance of these findings and assess whether music stimulation leads to measurable improvements in consciousness.

Finally, while we focused on the influence of sound frequency, other factors such as sound intensity or duration may also play a critical role in modulating cognitive and motor outcomes, which warrants further investigation.

## Conclusion

This study contributes to our understanding of the effects of various musical frequencies on the recovery of brain networks in patients with impaired consciousness. The observed changes in WA and frequency-specific coupling parameters emphasize the need to consider individual differences and specific frequency ranges when designing music-based interventions for neurorehabilitation objectives. Future research should investigate the long-term effects of music interventions, investigate the neural mechanisms underlying frequency-specific responses, and include additional outcome measures in order to gain a comprehensive understanding of the cognitive, emotional, and motor aspects of recovery in patients with impaired consciousness.

## Data Availability

The original contributions presented in the study are included in the article/supplementary material, further inquiries can be directed to the corresponding authors.
